# Acute Abdominal Pain Caused by an Infected Mesenteric Cyst in a 24-Year-Old Female

**DOI:** 10.1155/2016/8437832

**Published:** 2016-04-12

**Authors:** Davy R. Sudiono, Joep B. Ponten, Frank M. Zijta

**Affiliations:** ^1^Department of Radiology, Medical Center Haaglanden, Lijnbaan 32, 2512 VA Den Haag, Netherlands; ^2^Department of Surgery, Medical Center Haaglanden, Lijnbaan 32, 2512 VA Den Haag, Netherlands

## Abstract

A mesenteric cyst is a rare cause for abdominal pain. This umbrella term includes cystic entities which reside in the mesentery. We present a case of an infected false mesenteric cyst in a 24-year-old female patient without prior surgery or known trauma. Mainstay of treatment involves surgical resection, although less invasive treatments have been described. Prognosis depends on the origin of the cyst.

## 1. Introduction

The mesenteric cyst is a rare entity. It has been reported as an umbrella term that comprises any cystic mass which manifests within the mesentery, including lymphangioma, benign and malignant cystic lymphangioma, enteric duplication cyst, dermoid cyst, and pseudocyst [1]. Mesenteric cysts are often asymptomatic; however, if infected or ruptured patients may present with acute abdominal pain. This lack of specific symptoms and moreover its rare occurrence make diagnosis and consequently clinical decision challenging. Ultrasound (US) and contrast-enhanced computed tomography (CT) are decisive in distinguishing a wide variety of potential disorders that may result in acute abdominal pain. In case of a symptomatic mesenteric cyst a complete enucleation or resection either with laparoscopy or with laparotomy surgery is considered treatment of choice [2, 3]. We report a case of an infected mesenteric cyst in a 24-year-old woman presenting with severe abdominal pain and signs of infection, without previous abdominal surgery. Additionally we discuss relevant literature regarding histopathological differentiation and categorization as well as potential treatment options.

## 2. Case Presentation

A 24-year-old female without previous abdominal surgery or relevant medical history presented at her general practitioner because of severe abdominal pain, principally in the right lower quadrant, gradually increasing over the last 4 days. In addition she complained about lower back pain and feeling feverish. Consequently, she was admitted to the emergency department for advanced assessment. On physical examination at the time of admission, the patient experienced severe pain if palpitated in the right lower abdomen. Her vital signs were stable and cardiac and pulmonary examination were normal. She was afebrile; however, this could also be attributed to the use of anti-inflammatory drugs (NSAIDs). Laboratory results confirmed inflammation with an elevated C-reactive Protein (CRP) of 162 mg/L and a normal leucocytes amount of 9.8 × 10^9^/L. Urinalysis was normal. As a result acute appendicitis was suspected and imaging evaluation was performed.

**Figure 1:**
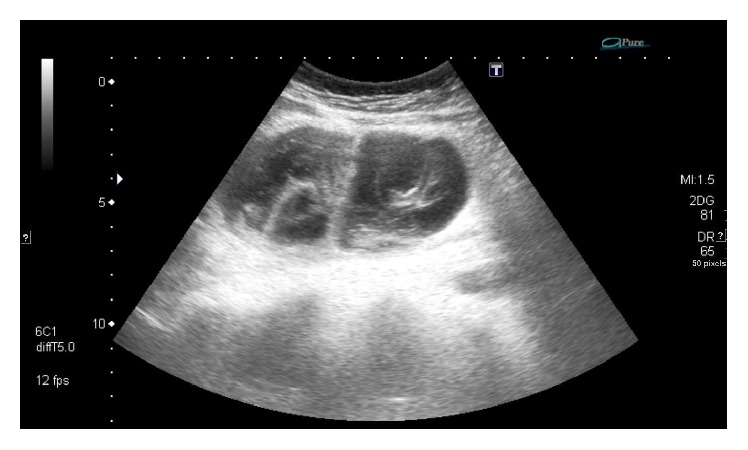
Ultrasound demonstrated a large abdominal, multilocular cystic mass. Notice the thick septations and echogenic content suspicious of a complicated cyst.

**Figure 2:**
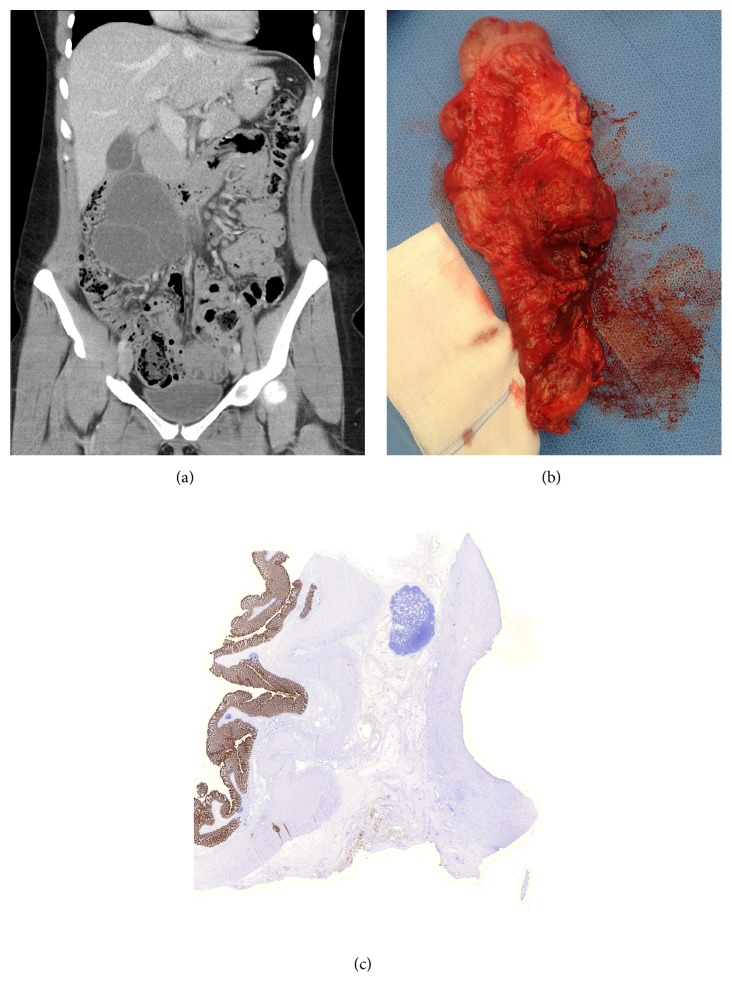
Mesenteric cyst in coronal plane in different viewings. (a) Contrast-enhanced CT. Cystic mass in close relation with surrounding bowel structures. (b) Macroscopic appearance of the resected ascending colon and adjacent mesenteric cyst in the mesenteric fat. (c) Histological appearance of the resected colon (left) and cyst (right). Notice the mesothelial cell specific AE1/AE3 positive (brown colour) cells lining the colon wall, which lacks at the cystic site. This confirms the lack of mesothelial cells in the cyst wall. AE1/AE3 stain, ×10.

Ultrasound revealed a large, moderately anechoic, multilocular mass in the right hemiabdomen with echogenic components extending from the caudal border of the liver into the right lower quadrant (Figure 1). Due to difficulties in defining the origin of the cystic mass, a complementary contrast-enhanced CT of the complete abdomen was performed. CT confirmed a well-encapsulated, intramesenteric, multilocular cystic mass in close proximity to the ascending colon, inferior part of the duodenum, and segmental mesenteric vessels (Figure 2(a)). The cystic mass with a mean CT-attenuation value of 10 Hounsfield Units (HU) showed no enhancing, solid components. The superior border of the mass showed distinctive, perifocal fat stranding. In accordance with ultrasound findings, a normal appearing right ovary was observed in the right lower abdomen, as well as a normal appearing appendix. Consequently, based on the radiologic findings an infected mesenteric cyst was suggested.

The patient retained severe, unceasing abdominal pain; consequently laparotomy was proposed. At surgery, enucleation of the cystic mass from the mesentery was attempted; however, due to the intraoperative findings of enlarged lymph nodes and the diffuse mesenteric infiltration in close relationship with adjacent ascending colon, an ileocecal resection was performed. Intraoperatively the cyst was partially opened and did not contain pus but rather greasy, brown, hemorrhagic-like material (Figure 2(b)). Macroscopically there seemed to be no relation with any muscular layer of the bowel wall. Fluid material was not sent for examination; therefore, bacterial growth was not determined.

**Figure 3:**
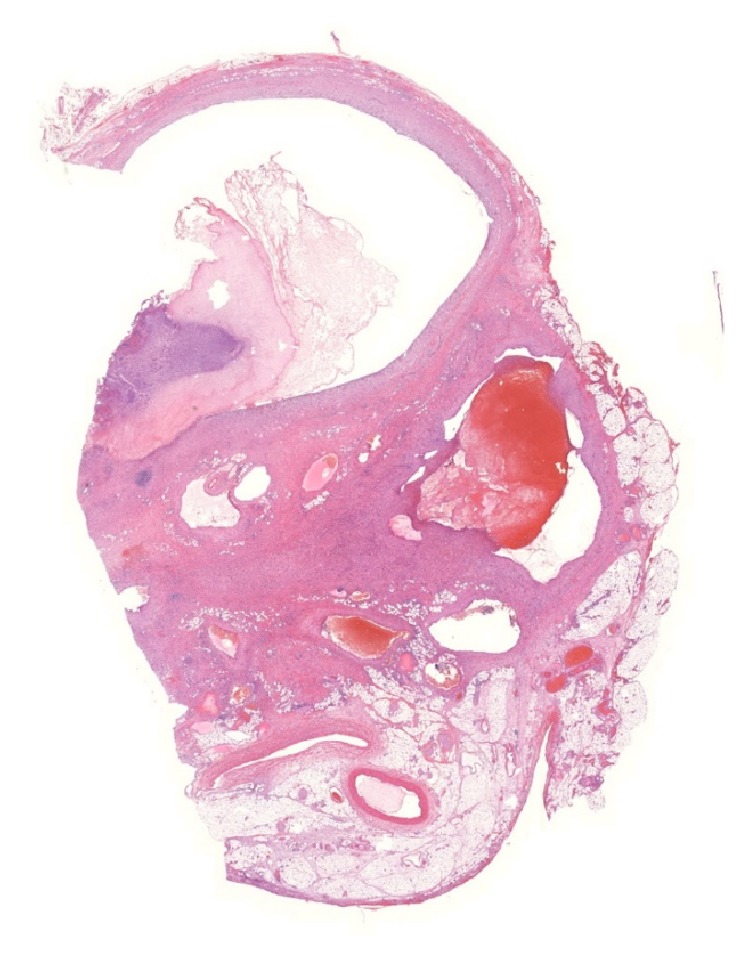
Histological image shows multiple smaller cysts spread out through the mesenteric fat. These represent areas of fat necrosis, which favor the diagnosis of a false cyst. Hematoxylin and eosin stain, ×10.

Microscopic examination of the specimen showed lining of the cystic wall with a flatted cell layer. Additional staining with mesothelial markers and endothelial markers was negative (Figure 2(c)). The peripheral border of the mass contained a fibrous moderately cell-rich wall with inflammatory, infiltrating changes. The cystic fluid consisted of protein rich fluids, combined with fibrin, inflammatory cells, and blood components. Furthermore multiple smaller cysts were noted in the mesenteric fat (Figure 3). Based on these findings a false infected mesenteric cyst was histopathologically confirmed. The patient recovered uneventfully and was discharged in good health three days after surgery.

## 3. Discussion

Mesenteric cysts are one of the rarest abdominal tumors with less than thousand cases reported in literature, with a reported incidence rate of approximately 1/100.000 admissions in adults [2, 4]. They seem to occur slightly more frequently in females and are found in almost all age groups with the highest incidence in the fourth decade of life [5]. Mesenteric cysts are often asymptomatic and are frequently incidental findings in routine abdominal imaging studies. However, if acute symptoms occur, that is, in case of complications, surgical resection is often required [5].

Symptoms are variable and rather nonspecific in case of a complicated cyst. Complications include infection, rupture, hemorrhage, and torsion. If large, the cyst can cause intestinal obstruction or bowel ischaemia on account of bowel compression or arterial compression, respectively [6, 7]. Although very rare, malignant degeneration has been described [7].

Ultrasound and contrast-enhanced abdominal CT are crucial in the diagnostic workup. The US appearance is reported to be diverse but should be considered if an avascular oval mesenteric mass is visualized [8]. CT is helpful for detecting signs of infection, rupture, or internal bleeding: a thickened enhancing cyst wall with perifocal fatty stranding is suggestive of such complications. On the other hand, US is superior to CT in demonstrating the internal nature of the cyst. Echogenic content, a thickened capsule, and septations indicate hemorrhage or infection [9].

Mesenteric cysts can occur anywhere in the mesentery along the gastrointestinal tract but most frequently appear in the small bowel mesentery, particularly the ileum [10]. Nonetheless determining the origin of an abdominal cystic structure remains a diagnostic challenge, whereas parapancreatic cysts and ovarian related pathology (e.g., ectopic endometriosis) should be ruled out. In addition, a fine needle aspiration has been reported to be helpful in distinguishing cysts of different origin [11]. MRI might be complementary in the characterization of the cysts content and its origin. As an alternative, preoperative laparoscopy could also be helpful in localizing and characterizing.

Numerous classifications have been reported to categorize mesenteric cysts based on pathology and etiologic origin [12, 13]. In 2000 de Perrot et al. proposed a classification based on histopathology [1], where distinction has been made between true and false cysts or pseudocysts. True cyst possesses an endothelial or mesothelial lining located on the inner wall, whereas a false cyst does not. In case of an endothelial inner lining it is classified as a cyst of lymphatic origin. On the other hand, a mesothelial cell lining defines a cyst of mesothelial origin. Hence, immunohistological analysis helps differentiating between these endothelial and mesothelial cells [14]. If the cyst arises from adjacent bowel wall, it is consequently classified as an enteric cyst or enteric duplication cyst. Lastly, cysts of urogenital origin and mature, cystic teratomas are the residual classes in this classification system [1].

Although the exact etiology is uncertain, an accepted theory for cysts of lymphatic origin entails a congenital benign proliferation of ectopic lymphatic tissue in the mesentery, which does not communicate accurately with the residual lymphatic system. By contrast, mesothelial cysts are believed to be a result of congenital failure of the mesenteric leaves to fuse [14]. False cysts are caused by trauma or other injuries like previous abdominal surgery or infection. In our case patients' medical history was unremarkable, in particular no history of previous abdominal surgery or trauma. Still the cyst was classified as a false cyst based on the lack of an endothelial or mesothelial cell lining. Furthermore, the presence of multiple smaller cysts in the mesentery indicated areas of fat necrosis supporting the classification of a false cyst. A report by Kim et al. [15] presented a similar case of a young female with an infected false mesenteric cyst without a history of predisposing factors.

Treatment of choice for complicated mesenteric cysts is surgery with complete resection of the cystic mass. This can be accomplished by either laparotomy or laparoscopy. An advantage of laparoscopy includes the shorter hospital stay but comes with longer procedure time and technical difficulty when the cyst is large or if infected [3]. In such cases laparotomy is advised. Depending on the nature of the cyst, anatomic relationships, and intraoperative findings, partial bowel resection might be necessary. This is mainly required when severance of important arteries in the mesentery cannot be avoided [16]. Simple aspiration or drainage is not advised due to a high recurrence rate and the chance of infection [2]. Three case reports have described a successful attempt to sclerose the cyst with ethanol after percutaneous drainage by the interventional radiologist [17, 18]. Complete regression of the cyst maintained after follow-up of up to 16 months. Although promising, this technique should be reserved for simple unilocular cysts, with no signs of malignant transformation.

Prognosis depends largely on the nature of the mesenteric cyst. Most are benign and therefore have a general good prognosis. Even so, mesotheliomas and lymphangiomas especially have a tendency to recur if resected incompletely [1].

## 4. Conclusion

Mesenteric cysts are rare abdominal tumors and encompass cysts from different origins with different etiologies. Consequently they can occur at diverse sites in the abdominal cavity. Symptomatic mesenteric cysts are preferably treated by surgical resection, through either laparotomy or laparoscopy. Also sclerosis of the cyst might be a feasible alternative, though with limitations. Diagnosis might be challenging and is mainly obtained with CT and US; therefore, radiologists should be aware of the existence of the (complicated) mesenteric cyst.
